# A Red-Emitting Fluorescence Sensor for Detecting Boronic Acid-Containing Agents in Cells

**DOI:** 10.3390/s22197671

**Published:** 2022-10-10

**Authors:** Naoya Kondo, Erika Aoki, Shinya Takada, Takashi Temma

**Affiliations:** Department of Biofunctional Analysis, Graduate School of Pharmaceutical Sciences, Osaka Medical and Pharmaceutical University, 4-20-1 Nasahara, Takatsuki 569-1094, Osaka, Japan

**Keywords:** fluorescence sensor, boron neutron capture therapy, boronic acid compounds, 4-borono-l-phenylalanine

## Abstract

The amount and localization of boron-10 atoms delivered into tumor cells determines the therapeutic effect of boron neutron capture therapy (BNCT) and, consequently, efforts have been directed to develop fluorescence sensors to detect intracellular boronic acid compounds. Currently, these sensors are blue-emitting and hence are impracticable for co-staining with nucleus staining reagents, such as DAPI and Hoechst 33342. Here, we designed and synthesized a novel fluorescence boron sensor, BS-631, that emits fluorescence with a maximum emission wavelength of 631 nm after reaction with the clinically available boronic acid agent, 4-borono-l-phenylalanine (BPA). BS-631 quantitatively detected BPA with sufficiently high sensitivity (detection limit = 19.6 µM) for evaluating BNCT agents. Furthermore, BS-631 did not emit fluorescence after incubation with metal cations. Notably, red-emitting BS-631 could easily and clearly visualize the localization of BPA within cells with nuclei co-stained using Hoechst 33342. This study highlights the promising properties of BS-631 as a versatile boron sensor for evaluating and analyzing boronic acid agents in cancer therapy.

## 1. Introduction

There is growing interest in boronic acid compounds that are not only used as medicinal chemistry intermediates [1] but also as therapeutic agents [2,3,4,5]. Furthermore, boronic acid compounds are particularly noteworthy as agents for boron neutron capture therapy (BNCT). BNCT is a cancer treatment method based on the nuclear reaction between boron-10 (10B) atoms delivered into cancer cells and externally irradiated thermal neutrons [6,7]. Therefore, it is reasonable to design compounds containing a boronic acid moiety to use as BNCT agents. The most widely used agent for BNCT is 4-borono-L-phenylalanine (BPA) [8], in which a boronic acid group replaces the hydroxy group of L-tyrosine [9]. In Japan, 4-BPA was marketed under the name Borofalan (Steboronine^®^) in May 2020 for locally advanced or recurrent unresectable head and neck cancer [10]. Several studies are now underway to expand the applicability of BPA for treatment of other cancer types and to develop novel BNCT agents whose physiological properties differ from those of BPA [11,12].

The nuclear reaction of the ^10^B atoms determines the therapeutic effect of BNCT; therefore, quantifying the amount of 10B in the tumor cells and analyzing the intracellular localization are critical steps in developing BNCT agents [13]. Inductively coupled plasma optical emission spectrometry and inductively coupled plasma mass spectrometry are the most widely used methods for quantifying boron amounts in tissues and cells. However, these require the combustion of the samples and are inappropriate for determining the intracellular localization of boron. Although imaging mass spectrometry using secondary ion mass spectrometry (SIMS) has been reported to evaluate intracellular boron localization [14,15], this approach lacks versatility because of the requirements for complex pre-treatments and expensive equipment.

Fluorescence sensors may be used to evaluate the intracellular localization of boron, and DAHMI has been adopted for this purpose for several years [12,16,17]. Recently, we developed a novel sensor, PPN-1 that has improved reactivity compared to that of DAHMI [18]. The treatment effect of BNCT is due to direct collisions between DNA and short-range high-energy particles (alpha particles and Li nuclei) that result from the nuclear reaction of 10B atoms with irradiated thermal neutrons and create double strand breaks in DNA [19]. Therefore, nuclei co-staining is also required to determine the subcellular localization of the boron agents. The boron sensors developed so far, such as DAHMI, emit blue fluorescence after reacting with boronic acid compounds. Consequently, these require unusual staining reagents that emit green or red fluorescence for the nucleus co-staining instead of the commonly used DAPI or Hoechst 33342, both of which emit blue fluorescence. A GFP-based reagent has been described for green fluorescence nucleus staining [17] but is expensive and extensive time is required for staining. Such protein-based dyes require a membrane permeabilization process beforehand, which may alter the localization and amount of boron agents in cells. To address these shortcomings, we sought to develop a small molecule fluorescence boron sensor with a longer emission wavelength that can be co-stained with typical nucleus-staining reagents, such as DAPI and Hoechst 33342.

**Figure 1:**
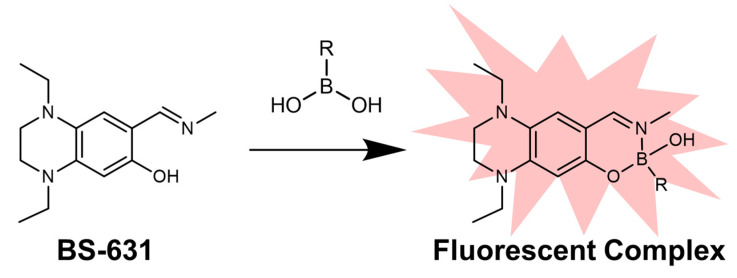
Schematic of boronic acid detection by fluorescent complex with BS-631.

Therefore, we designed BS-631 (Figure 1) by integrating a tetrahydroquinoxaline (TQ) moiety and 2-(iminomethyl)phenol with an N, O-chelating ligand substructure. The N, O-chelating ligand is known to form a stable complex with boronic acid compounds in a specific manner, making it a practical basic framework for fluorescent boron sensors [16,20,21]. TQ is a potent electron donor that promotes intramolecular charge transfer. We thus expected that introducing the TQ moiety into the N, O-ligand would extend the emission wavelength [22,23,24]. In this study, we synthesized BS-631 and evaluated the fluorescence properties and potential use in visualizing the subcellular localization of BPA.

## 2. Materials and Methods

### 2.1. Preparation of BS-631

All reagents were obtained commercially and used without further purification. BPA (^10^B) was supplied from Stella Pharma Corp. (Osaka, Japan). Synthesis of the intermediates was performed as previously described [23,25] with minor modifications. The synthetic scheme is shown in Figure S1 and the details are described in the Supplementary Methods. Purified BS-631 was obtained as a brown oil and characterized using an EI-MS, a high-resolution mass spectra (HRMS)(JMS-700(2) mass spectrometer, JEOL Ltd., Tokyo, Japan), and ^1^H and ^13^C-NMR (DD2 NMR Spectrometer, Agilent, CA, USA, 600 MHz). MS (EI) *m*/*z*: 247; EI-HRMS *m*/*z*: 247.1681 (chemical formula: C_14_H_21_N_3_O, calculated *m*/*z*: 247.1685); ^1^H NMR (600 MHz, CDCl_3_): δ 7.76 (s, 1H), 6.18 (s, 1H), 6.04 (s, 1H), 3.44 (t, *J* = 5.0 Hz, 2H), 3.35 (q, *J* = 7.0 Hz, 2H), 3.29 (s, 3H), 3.20 (q, *J* = 7.0 Hz, 2H), 3.11 (t, *J* = 5.0 Hz, 2H), 1.17 (t, *J* = 5.0 Hz, 3H), and 1.16 (t, *J* = 5.0 Hz, 3H); ^13^C NMR (600 MHz, CDCl_3_): δ 165.0, 162.2, 143.0, 127.1, 112.0, 107.4, 98.5, 47.5, 45.6, 45.6, 45.4, 41.8, 10.6, and 10.3.

### 2.2. Fluorescence Property

BS-631 was mixed with BPA to final concentrations of 100 μM and 1.0 mM, respectively, in 0.5% DMSO/H_2_O. Excitation and emission spectra were measured at 60 min using a spectrometer (FP-8600, JASCO Corporation, Tokyo, Japan, photomultiplier voltage: 1200 V) to evaluate the maximum excitation ($\lambda_{\max}^{ex}$) and emission ($\lambda_{\max}^{em}$) wavelengths. The absorption spectrum of BS-631 (50 μM) was measured with a UV-visible spectrophotometer (V-730, JASCO) after 60 min incubation with BPA (final concentration of 0.5 mM) in 0.5% DMSO/H_2_O. To examine time-dependent changes in fluorescence, the same measurements were performed at 30 min and 120 min.

To evaluate the relationship between the fluorescence intensity and BPA concentration, BS-631 was mixed to a final concentration of 100 μM with various concentrations of BPA (0–1000 μM at final concentration) in 0.5% DMSO/H_2_O (*n* = 3). Fluorescence intensities were measured 60 min after mixing using a plate reader (EnSpire Multilabel Reader 2300, PerkinElmer Japan, Kanagawa, Japan, λex/λem: 430/630 nm). A linear regression analysis was performed to fit the data and calculate detection and quantification limits from the following equations: detection limit = 3 σ/slope and quantification limit = 10 σ/slope (where σ is the standard deviation of the fluorescence intensities of samples at 0 µM, and the slope is derived from the regression line.)

### 2.3. Selectivity Assay

BS-631 (final concentration of 100 μM) was mixed with BPA or a metal cation (NaCl, MgCl_2_·6H_2_O, KCl, CoCl_2_·6H_2_O, or NiCl_2_·6H_2_O) (final concentration of 1.0 mM) in 0.5% DMSO/PBS(-) (pH = 7.4). Fluorescence intensities of the samples were measured 60 min after mixing using a plate reader (λex/λem: 430/630 nm) in triplicate (*n* = 3). These fluorescence intensities were expressed as a relative value compared to the BS-631 solution in the absence of metal cations and BPA. AlCl_3_·6H_2_O, CaCl_2_, MnCl_2_·4H_2_O, FeCl_2_·4H_2_O, FeCl_3_·6H_2_O, CuCl_2_, ZnCl_2_, and CdCl_2_·2.5H_2_O were dissolved in 0.5% DMSO/Tris HCl buffer (100 mM, pH = 7.4) and measured in same manner.

### 2.4. Fluorescence Microscopy Study

T3M-4 human pancreatic adenocarcinoma cells (RCB1021) were provided by the RIKEN BioResource Research Centre (Ibaraki, Japan) and were cultured in RPMI 1640 containing 10% fetal bovine serum at 37 °C in a humidified atmosphere of 5% CO_2_. The uptake of BPA into T3M-4 cells was performed with several modifications to the previously reported method [26]. Briefly, T3M-4 cells were cultured in a 35 mm dish with a glass bottom (Matsunami Glass Ind., Osaka, Japan) 2 days before the experiment. After washing twice with Hank’s balanced salt solution (HBSS: 125 mM choline chloride, 4.8 mM KCl, 1.2 mM MgSO_4_, 1.2 mM KH_2_PO_4_, 1.3 mM CaCl_2_, 5.6 mM D-glucose, and 25 mM HEPES), 1 mL of BPA (1 mM in HBSS) was added and incubated at 37 °C for 10 min. For the BPA-absent group, only HBSS was added and incubated. After washing three times with PBS(-), cells were fixed with 4% paraformaldehyde for 30 min at room temperature and incubated for 30 min, followed by incubation with 1 mL of BS-631 (100 μM in 0.5% DMSO/PBS(-)) for 30 min at room temperature. For nuclei staining, cells were incubated with Hoechst 33342 (5 μg/mL, Nacalai Tesque, Kyoto, Japan) for 10 min at room temperature. Fluorescence images were acquired using a BZ-X810 (Keyence, Osaka, Japan) instrument.

### 2.5. Statistics

Data are presented as means ± standard deviation. Statistical analyses were performed using Dunnett’s multiple comparison tests or unpaired t-test using GraphPad Prism 8. Differences at the 95% confidence level (*p* < 0.05) were considered significant unless otherwise noted.

## 3. Results and Discussion

### 3.1. Fluorescence Properties of BS-631

**Figure 2:**
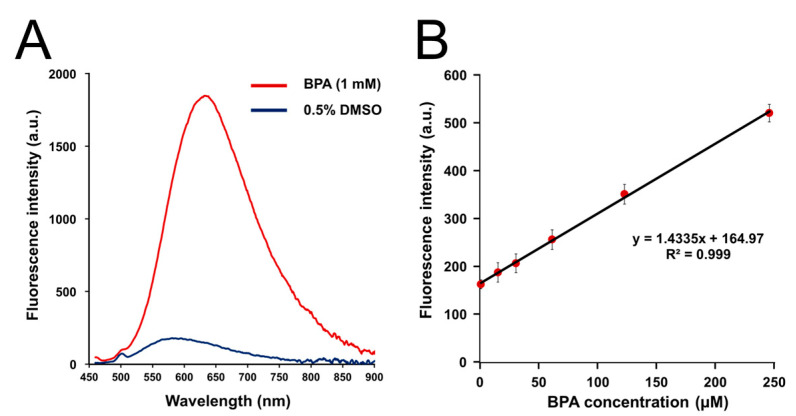
Increase in BS-631 fluorescence intensity in reaction with BPA. (**A**) Emission spectra of BS-631 (100 μM) 60 min after addition of BPA (0 or 1 mM) in 0.5% DMSO/H_2_O (λex = 430 nm). (**B**) Linear regression analysis between the fluorescence intensities of BS-631 (100 μM) and BPA (0–250 μM) in 0.5% DMSO/H_2_O (λex = 430 nm).

BS-631 was synthesized with a total yield of 3.2%. BS-631 was easily dissolved in 0.5% DMSO/PBS(-) at a concentration of 100 μM, and the ClogP value of 2.85 for BS-631 was comparable to that of DAHMI (2.81), suggesting that both have comparable water solubility and membrane permeability. After the additions of BPA, BS-631 emitted fluorescence at 425 and 631 nm for the maximum excitation ($\lambda_{\max}^{ex}$) and emission wavelengths ($\lambda_{\max}^{em}$), respectively (Figure S2 and Figure 2A). The absorption spectrum of BS-631 after incubation with BPA is shown in Figure S3. As expected, the excitation and emission wavelengths are longer than those of DAHMI ($\lambda_{\max}^{ex}$, 408 nm; $\lambda_{\max}^{em}$, 430 nm) [16], and the Stokes shift is considerably extended. The BS-631 fluorescence does not overlap with that of the typical nucleus staining reagents DAPI ($\lambda_{\max}^{ex}$, 358 nm; $\lambda_{\max}^{em}$, 461 nm) and Hoechst 33342 ($\lambda_{\max}^{ex}$, 361 nm; $\lambda_{\max}^{em}$, 497 nm). Fluorescence intensity increased at 60 min after mixing but decreased slightly at 120 min (Figure S4). This result may suggest the instability of BS-631 in an aqueous solution over a longer period of time.

Linear regression analysis revealed that the fluorescence intensities from the reaction of BS-631 (100 μM) with 0–250 μM BPA had high linearity (R^2^ = 0.999, Figure 2B). Furthermore, the limits of detection and quantification were determined as 19.6 and 65.3 µM, respectively. Since BNCT requires an intratumor boron concentration of more than 20 ppm [7], equivalent to 2 mM BPA, we considered the detection and quantification limits of BS-631 to be sufficiently high enough for evaluation of BNCT agents. The linearity was maintained up to 500 µM BPA (R^2^ = 0.999, Figure S5) but saturation was observed at 1000 µM (Figure S6). Therefore, linearity is thought to be maintained up to five times the concentration of the BPA compared to the sensor.

### 3.2. Selectivity Assay

**Figure 3:**
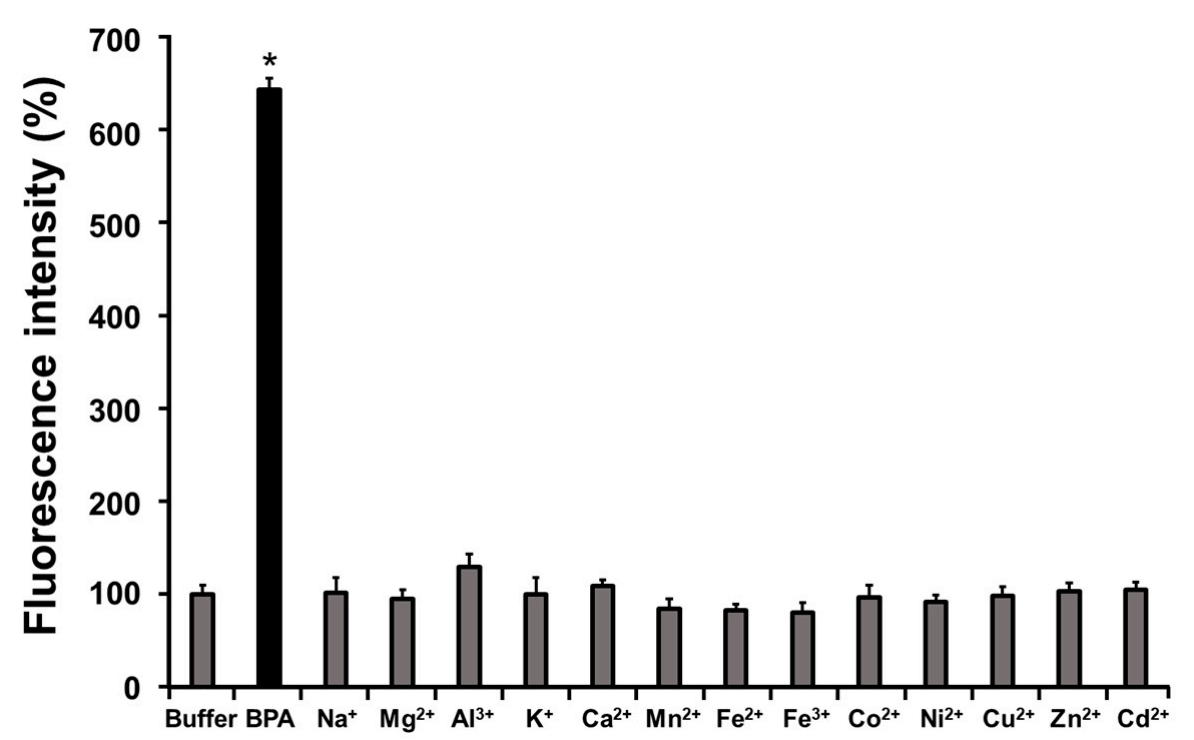
BS-631 fluorescence intensity (100 μM) 60 min after addition of BPA or metal cation (1.0 mM, pH = 7.4) * *p* < 0.05 vs. buffer (PBS(-) only) by Dunnett’s multiple comparison test.

The changes in the fluorescence of BS-631 (100 μM) after the addition of various metal cations (1.0 mM) are summarized in Figure 3. There was no significant increase in the fluorescence of BS-631 following incubation with any of the metal cations tested. However, there was a significant increase in the fluorescence of BS-631 (>600%) following incubation with BPA. These results suggest that BS-631 is more reactive with boronic acid than other metal cations.

### 3.3. Fluorescence Microscopy Study

**Figure 4:**
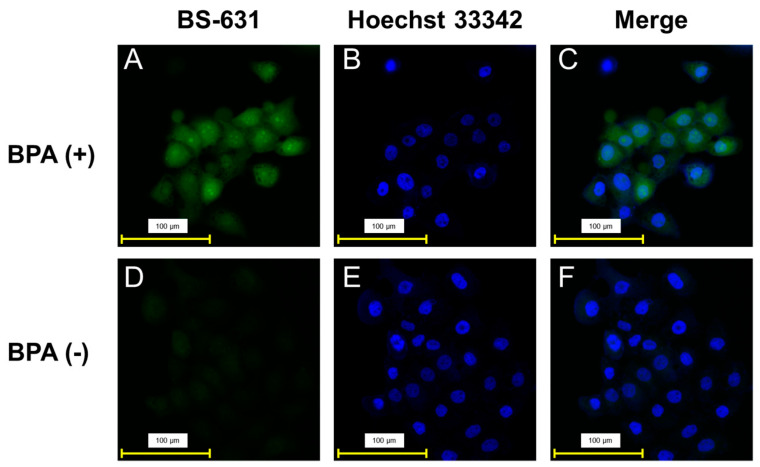
Representative fluorescence images of T3M-4 cells: (**A**–**C**) BPA-present group, (**D**–**F**) BPA-absent group. (**A**,**D**) Fluorescence images after 30 min incubation with BS-631 (100 μM) (GFP filter: Ex: 470/40 nm, Em: 525/50 nm). (**B**,**E**) Nucleus staining using Hoechst 33342 (DAPI filter: Ex: 360/40 nm, Em: 460/50 nm). (**C**,**F**) Merged images of fluorescence from BS-631 and Hoechst 33342.

Fluorescence images were filtered using a GFP filter cube (Keyence OP-87763, Ex: 470/40 nm, Em: 525/50 nm, pseudo-color: green) after the addition of BS-631 to cells with or without BPA, as shown in Figure 4. Although faint fluorescence was observed in the BPA-absent group (Figure 4D), fluorescence was observed throughout cells in the BPA-treated group (Figure 4A) and was slightly stronger around the cell nuclei. This result is consistent with previously reported evaluations of intracellular localization evaluation using SIMS [14,15]. In addition, close observation of the images revealed strong fluorescence in areas that appeared to be the cell nucleoli. Although currently used fluorescence sensors cannot unveil the microscopic localization of BPA, BS-631 can provide clear images that allow localization observation at the level of cellular organelles.

Co-staining of boron localization with BS-631 and nucleus staining with Hoechst 33342 (Figure 4B,C,E,F) was feasible because the fluorescence derived from Hoechst 33342 was filtered using a DAPI filter (Keyence OP-87762, Ex: 360/40 nm, Em: 460/50 nm, pseudo-color: blue) and did not overlap with that of BS-631. The merged images also implied slightly higher boron accumulation in the nucleolus. While the commercially available GFP filter used in this experiment could provide fluorescent images from BS-631, a filter set to be optimized for the maximal wavelengths of BS-631 would improve the image quality and the usability of the sensor in the future.

BS-631 visualization was not possible in cells treated with Triton X for cellular membrane permeabilization after BPA accumulation (data not shown), suggesting that the permeabilization procedure washed the accumulated BPA from the cells. Therefore, we used Hoechst 33342 for the nucleus staining rather than DAPI, which has low membrane permeability and requires permeabilization treatment. BS-631 does not require membrane permeabilization to visualize intracellular boronic acid compounds, probably due to its membrane-permeable property. Although there is possible concern about the stability of BS-631 in aqueous solution for long periods of time, it is not expected to affect the detection of BS-631 for short periods of time, such as 30 min. In addition, no obvious cytotoxicity was observed in our preliminary experiments with living cells. Future application of BS-631 to unfixed live cells may require detailed cytotoxicity evaluation using other methods, such as MTT assay and WST assay, as well as investigation into the effect of BS-631 on the accumulation of boronic acid compounds in the cells.

In this study, we incubated cells with BPA, the only available BNCT agent in clinical use, and successfully visualized its subcellular localization. Theoretically, other boronic acid-containing agents may emit fluorescence by the same mechanism, and we believe that BS-631 can be applied to other boronic acid agents, but the actual applicability of this technique remains to be demonstrated in the future.

## 4. Conclusions

We developed a fluorescence sensor, BS-631, that reacts with BPA and emits red fluorescence to quantitatively detect BPA with sufficiently high sensitivity. BS-631 easily and clearly visualized the intracellular localization of BPA with the co-stained cell nuclei co-stained using Hoechst 33342. This study thus highlights the promising properties of BS-631 as a versatile boron sensor for evaluating and analyzing future BNCT agents.

## Data Availability

The data supporting the results and findings of this study are available within the paper and the Supplementary Information files. Additional raw data are available from the corresponding author upon request.

## Supplementary Materials

The following supporting information can be downloaded at: https://www.mdpi.com/article/10.3390/s22197671/s1, Figure S1: Synthetic scheme of BS-631; Figure S2: Excitation and emission spectra of BS-631 at 60 min after addition of BPA; Figure S3: Absorption spectrum of BS-631 (50 μM) at 60 min after addition of BPA; Figure S4: Emission spectra of BS-631 at 30, 60, and 120 min after addition of BPA; Figure S5: Linear regression analysis between the fluorescence intensities of BS-631 (100 μM) and BPA (0–500 μM); Figure S6: Linear regression analysis between the fluorescence intensities of BS-631 (100 μM) and BPA (0–1000 μM), and Supplementary Methods.
