# Supplementary material for: A Red-Emitting Fluorescence Sensor for Detecting Boronic Acid-Containing Agents in Cells
Source: Sensors (Basel). 2022 Oct 10;22(19):7671. doi: 10.3390/s22197671 (PMC9573690; doi:10.3390/s22197671)
Supplement: Supplementary file 1 [file sensors-22-07671-s001.zip › sensors-1902458-supplementary.pdf]

Supplementary information

A red-emitting fluorescence sensor for detecting boronic acid-containing agents in cells

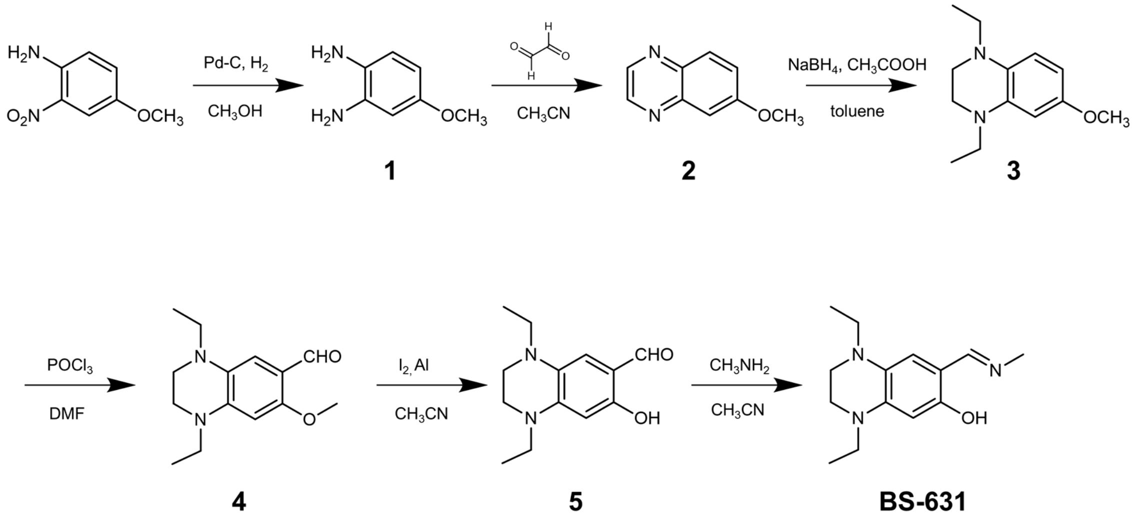

Figure S1

Synthetic scheme of BS-631

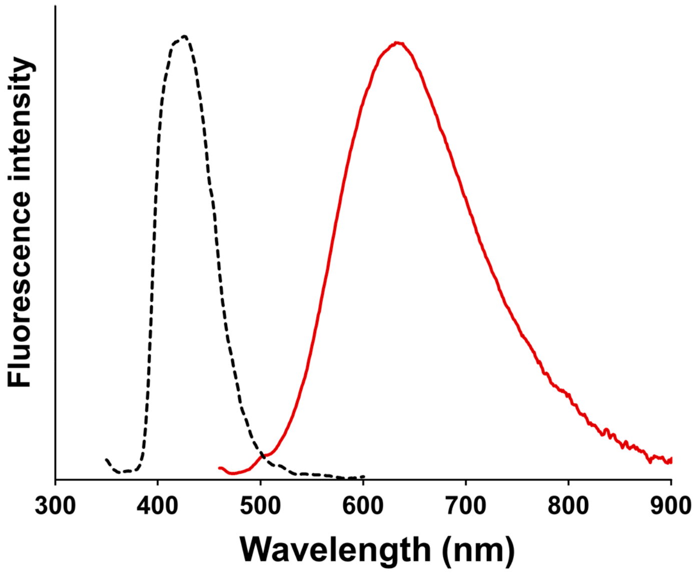

Figure S2

Excitation (dotted black line, λem = 630 nm) and emission spectra (red line, λex = 430 nm) of BS-631 (100 µM) at 60 min after addition of BPA (0 or 1 mM) in 0.5% DMSO/H_2O.

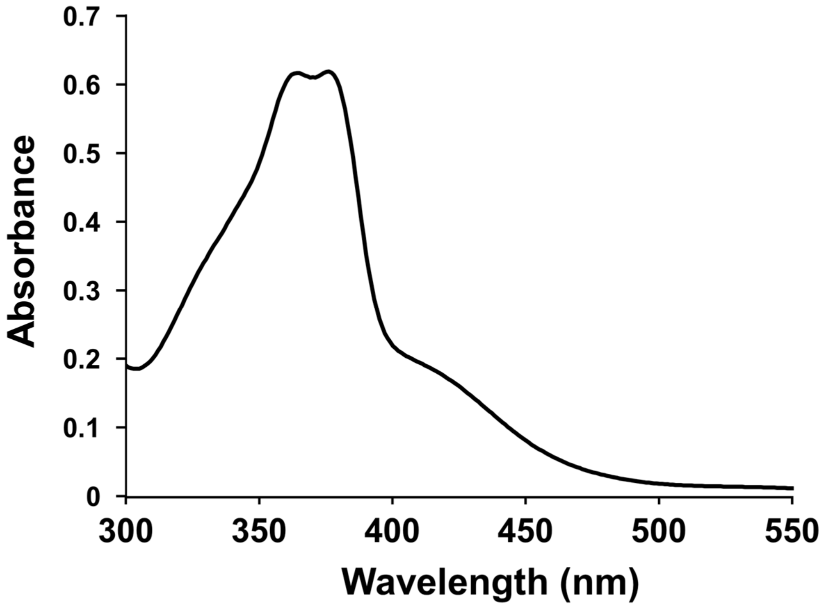

Figure S3

The absorption spectrum of BS-631 (50 μM) at 60 min addition of BPA (0.5 mM final concentration) in 0.5% DMSO/H_2O.

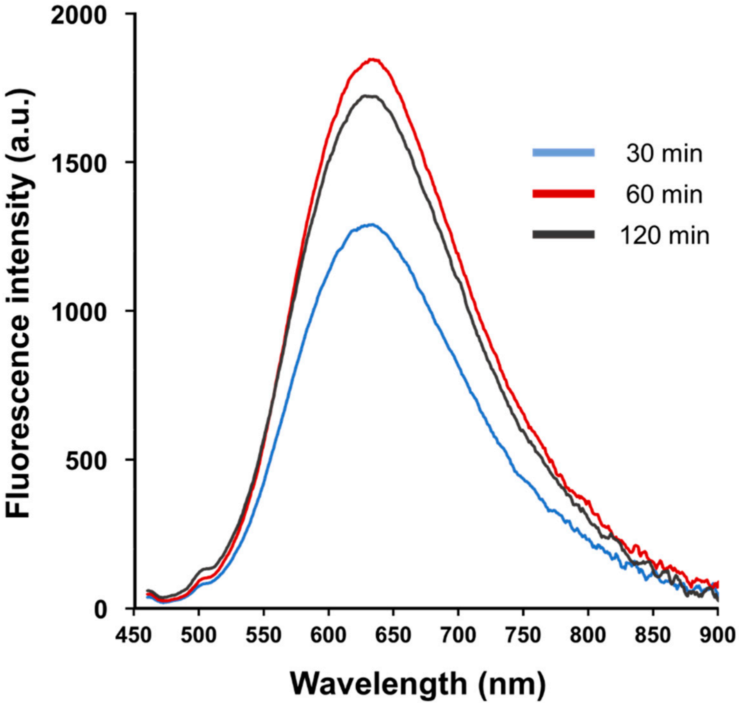

Figure S4

Emission spectra of BS-631 (100 μM) at 30, 60, and 120 min after addition of BPA (1 mM) in 0.5% DMSO/H_2O (λex = 430 nm).

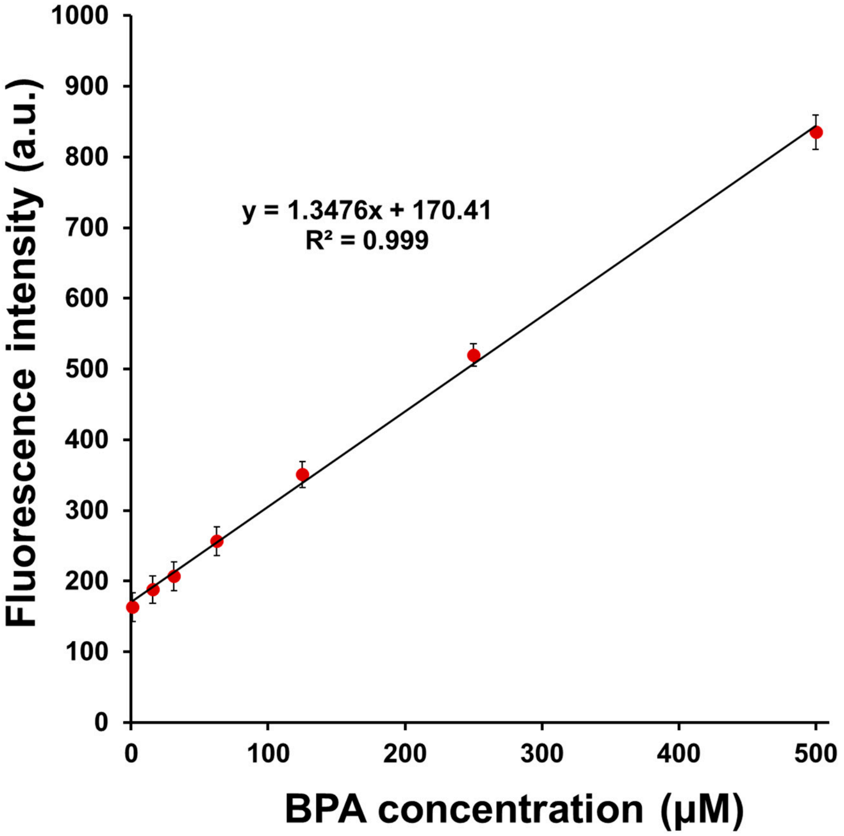

Figure S5

Linear regression analysis between the fluorescence intensities of BS-631 (100 μM) and BPA (0–500 μM) in 0.5% DMSO/H_2O (λex = 430 nm).

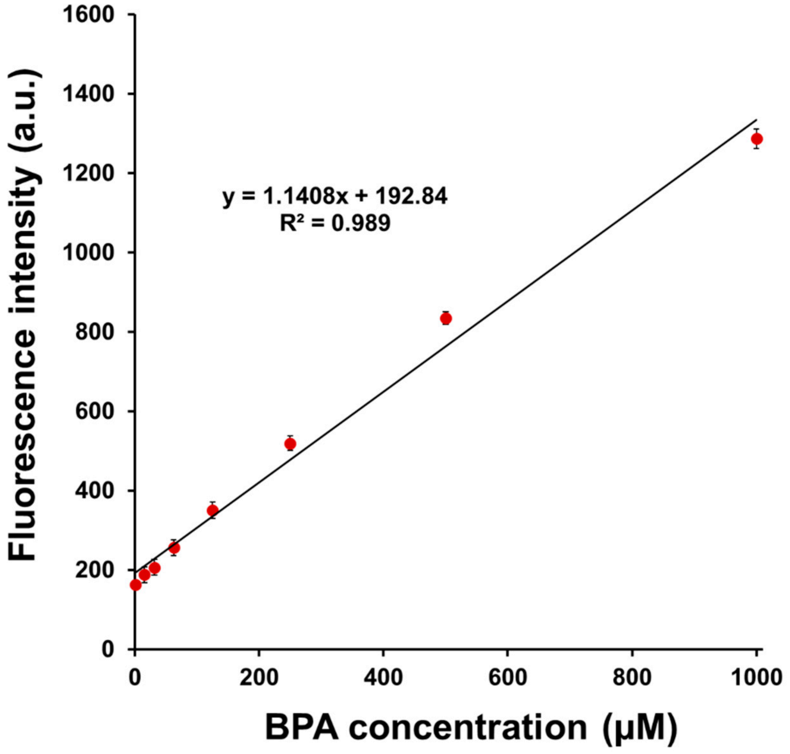

Figure S6

Linear regression analysis between the fluorescence intensities of BS-631 (100 μM) and BPA (0–1000 μM) in 0.5% DMSO/H_2O (λex = 430 nm).

Supplementary Methods

General materials and methods for synthesis

All chemical reagents and solvents were obtained from commercial suppliers and used without further purification. Thin layer chromatography (TLC) was performed on silica gel 60 F254 precoated aluminum sheets (Sigma-Aldrich Japan, Tokyo, Japan) and visualized by UV-light. Chromatographic purification was accomplished using a flash

column chromatography system (Isolera Four, Biotage Japan Ltd., Tokyo, Japan) equipped with silica gel flash cartridges (Rening series, Biotage Japan). ^{1}H- and ^{13}C-NMR spectra were recorded in deuterated solvents on a Varian Mercury 400 (400 MHz) spectrometer or DD2 NMR Spectrometer (600 MHz, Agilent, CA, USA), calibrated to tetramethylsilane (= 0 ppm). Multiplicities are abbreviated as follows: s = singlet, d = doublet, t = triplet, q = quartet, m = multiplet, dd = double doublet.

4-methoxybenzene-1,2-diamine *(1)*

4-Methoxy-2-nitroaniline (2.10 g, 12.5 mmol) was hydrogenated in methanol (140 mL) with 10% Pd/C (1.9 g) at 40 °C for 3 h. The resulting solution was filtered through celite and concentrated by rotary evaporation to give 4-methoxybenzene-1,2-diamine ***(1)*** as a brown oil, which was used for the next step without further purification.

6-methoxyquinoxaline *(2)*

glyoxal solution (40% aqueous solution, 69 mmol) was added to the solution of compound **1** in acetonitrile (45 mL). The resulting mixture was stirred at 60 °C for 3 h. The reaction mixture was cooled to rt and concentrated under reduced pressure. the residue was purified by silica gel chromatography (Hexane/EtOAc = 100/0 to 60/40) to

afford ***(2)*** (1.01 g, 51%, two steps) as a light-yellow solid. ^{1}H NMR (400 MHz, $CDCl_3$): δ 8.74 (d, J = 2.0 Hz, 1H), 8.68 (d, J = 2.0 Hz, 1H), 7.97 (d, J = 9.2 Hz, 1H), 7.43 (dd, J = 9.2, 2.8 Hz, 1H), 7.36 (d, J = 2.4 Hz, 1H), 3.96 (s, 3H).

1,4-diethyl-6-methoxy-1,2,3,4-tetrahydroquinoxaline *(3)*

$NaBH_4$ (2.47 g, 65.3 mmol) was added in portions over a period of 15 min to the solution of compound **2** (1.01 g, 6.3 mmol) in 30 mL toluene at 0 °C , and the reaction mixture was stirred for 10 min. Next, acetic acid (10 mL) was added to the reaction mixture dropwise at 0 °C. The resulting mixture was stirred for 1 h at rt and then refluxed for another 5 h. After cooling to rt, water (80 mL) was added to the mixture to quench the reaction. The organic layer was separated, and the aqueous layer was extracted with ethyl acetate (3 x 50 mL). Then, obtained organic solution was washed with aqueous Na_2CO_3 solution and H_2O, and dried over anhydrous Na_2SO_4, and concentrated under reduced pressure. the residue was purified by silica gel chromatography (Hexane/EtOAc = 80/20 to 70/30) to afford ***(3)*** (1.08 g, 77%) as a light-brown oil. ^{1}H NMR (400 MHz, $CDCl_3$): δ 6.49 (d, J = 8.4 Hz, 1H), 6.15-6.20 (m, 2H), 3.74 (s, 3H), 3.21-3.36 (m 8H), 1.14 (t, J = 14.0 Hz, 6H).

1,4-diethyl-7-methoxy-1,2,3,4-tetrahydro-quinoxaline-6-carbaldehyde *(4)*

Phosphorous oxychloride (0.79 mL, 27.0 mmol) and DMF (0.98 mL, 38.0 mmol) were mixed at 5 °C and stirred for 15 min. Compound **3** (1.08 g, 4.9 mmol) in DMF (0.64 mL) was added to the mixture at 75 °C for 4 h. After cooling to rt, ice-cold water was poured into the mixture and then neutralized with cold NaOH solution (15%). The solution was extracted with ethyl acetate (4 x 50 mL). The organic layer was separated and dried over anhydrous Na_2SO_4 and concentrated under reduced pressure. the residue was purified by silica gel chromatography (Hexane/EtOAc = 80/20 to 50/50) to afford ***(4)*** (0.53 g, 43%) as an orange oil. ^1H NMR (400 MHz, $CDCl_3$): δ 10.12 (s, 1H), 6.96 (s, 1H), 6.03 (s, 1H), 3.86 (s, 3H), 3.51-3.53 (m, 2H), 3.44 (q, J = 21.2 Hz, 2H), 3.34 (q, J = 7.2 Hz, 2H), 3.15-3.17 (m, 2H), 1.23 (t, J = 14.4 Hz, 3H), 1.16 (t, J = 14.4 Hz, 3H).

1,4-diethyl-7-hydroxy-1,2,3,4-tetrahydro-quinoxaline-6-carbaldehyde *(5)*

To a slurry of aluminum (0.08 g, 3.0 mmol) in acetonitrile (2.2 mL) was added iodine (0.98 g, 7.7 mmol) in portions. Stir the reaction mixture under argon atmosphere till the color of the solution changed to yellow. Then, the solution of compound **4** (0.53 g, 2.3 mmol) in acetonitrile (1.2 mL) was added dropwise and the resulting mixture was then refluxed under argon atmosphere for 10 h. After cooling to rt, the reaction mixture

was poured into cold water (50 mL). Extract the mixture with ethyl acetate (4 x 50 mL) and combine the organic layer. The organic layer was washed with brine and dried over anhydrous Na_2SO_4 and concentrated under reduced pressure. the residue was purified by silica gel chromatography (Hexanes/EtOAc = 90/10 to 70/30) to afford ***(5)*** (0.29 g, 58%) as a dark yellow oil. ^{1}H NMR (400 MHz, $CDCl_3$): δ 11.58 (s, 1H), 9.45 (s, 1H), 6.48 (s, 1H), 6.04 (s, 1H), 3.52 (t, J = 10.0 Hz, 2H), 3.39 (q, J = 6.8 Hz, 2H), 3.28 (q, J = 14.4 Hz, 2H), 3.14 (t, J = 10.4 Hz, 2H), 1.17-1.22 (m, 6H).

BS-631

To a solution of compound 6 (290 mg, 1.24 mmol) in acetonitrile (60 mL) was added methylamine (1.24 mmol, 1 eq.), and refluxed for 18 h. The reaction mixture was concentrated under reduced pressure. the residue was purified by silica gel chromatography (Chloroform/MeOH = 100/0 to 80/20) to afford **BS-631** (100 mg, 33%) as a brown oil.
